# Effects of mulch films with different thicknesses on the microbial community of tobacco rhizosphere soil in Yunnan laterite

**DOI:** 10.3389/fmicb.2024.1458470

**Published:** 2024-09-23

**Authors:** Shuaibing Wang, Qiuping Li, Changbing Ye, Wenqing Ma, Yandong Sun, Bin Zhao, Weiqing Zeng, Zhiqiang Yue, Lan Li, Dandan Li

**Affiliations:** ^1^School of Chemistry, Biology and Environment, Yuxi Normal University, Yuxi, China; ^2^College of Agronomy and Biotechnology, Yunnan Agricultural University, Kunming, China; ^3^Agricultural Environmental Protection and Rural Energy Workstation, Yuxi Agriculture and Rural Bureau, Yuxi, China; ^4^School of Geography and Land Engineering, Yuxi Normal University, Yuxi, China

**Keywords:** mulch thickness, rhizosphere microorganism, soil characteristics, tobacco, interactions

## Abstract

The mulch film (MF) management model of the agricultural field affects the physical and chemical properties of soil (PCPS) and the structure of the microorganism community; however, studies on the relationship between the rhizosphere microorganism community structure and the thickness of MF are still limited. To understand the interactions among the MF thickness, PCPS, and rhizosphere microorganism, a study was conducted by using an integrated metagenomic strategy, where tobacco rhizosphere soil was treated with four commonly representative and used thicknesses of MFs (0.004, 0.006, 0.008, and 0.010 mm) in Yunnan laterite. The results showed that agronomic traits such as the tobacco plant height (TPH), leaf number (LN), fresh leaf weight (FLW), and dry leaf weight (DLW) were significantly (*p* < 0.01) improved in the field mulched with the thickest film (0.010 mm) compared with the exposed field (CK), and there was a 6.81 and 5.54% increase in the FLW and TPH, separately. The correlation analyses revealed a significant positive correlation of the MF thickness with the soil water content (SWC), soil organic matter (SOM), total nitrogen (TN), available nitrogen (AN), total phosphorus (TP), and available phosphorus (AP; all *p* < 0.01), while the MF thickness was negatively correlated with the soil temperature (ST; *p* < 0.01). In addition, the community structure of the rhizosphere soil bacteria was significantly changed overall by the MF thickness, which also interfered with the function of the rhizosphere soil bacteria. The correlation analyses also showed that the abundance of *Bradyrhizobium* and *Nitrospira* was positively correlated with the MF thickness, while the abundance of *Sphinsinomonas* and *Massilia* was negatively correlated with it. This indicated that with the increase of the MF thickness, the ability of the rhizosphere soil to utilize N and remove harmful molecules was strengthened, while the capacity of the rhizosphere soil to degrade pollutants was greatly reduced. These findings provide additional insights into the potential risks of the application of different thicknesses of MFs, particularly concerning the PCPS and soil microbial communities.

## 1 Introduction

Yunnan laterite used in tobacco production is very sticky, poorly developed, consists of low soil organic matter (SOM), and is prone to nutrient leaching (Barton et al., 2004; Duan et al., 2015; Liang et al., 2022). In addition, tobacco yield and quality are closely and positively correlated with soil productivity, much of which is caused by soil nutrient deficiency, a lower microbial metabolic activity (LMMA) growth rate, and a less flue-cured tobacco nutrient accumulation (Wang et al., 2019; Yan and Liu, 2020; Zhai et al., 2022; Chen et al., 2023). Furthermore, LMMA has also been shown to impair the root system of tobacco plants, leading to reduced biomass (Li et al., 2022). The majority of the plant biomass loss is due to poor water-holding capacity and nutrient uptake being affected by LMMA (Wang G.et al., 2023; Shang et al., 2023).

Biodegradable mulch (BM), as an environmentally friendly material, provides high water and nutrient retention, reduces the need for fertilizers, and improves crop yield and quality (Li et al., 2004; Harshitha et al., 2022; Yang et al., 2023). BMs also serve as a commonly applied soil improvement material in agricultural production, enhancing the soil quality by increasing the cation exchange soil bulk density (SBD), soil water content (SWC), SOM, and beneficial soil microorganisms (Huang et al., 2022; Kai et al., 2024). The application of BMs may prove useful as a management strategy for mitigating soil erosion while ameliorating the soil microbial community structure and metabolic activity (Sreejata et al., 2018). In a previous study, various factors, such as soil temperature (ST), moisture, and nutrients, of plant rhizosphere soil significantly changed due to the covering of the field with BMs (Gunina and Kuzyakov, 2022; Jiao et al., 2021). In another study, it was observed that the diversity and composition of the rhizosphere microorganism also changed thereupon in the field covered with BMs; after the change, the rhizosphere microorganism was found to be more conducive to carbon (C) and N mineralization and crop growth (Zhang et al., 2024b). In addition, the degradation of BMs considerably affect soil microbial communities by directly changing the soil micro-environment and indirectly altering the nutrient cycling and fate (Ding et al., 2021).

At present, some researchers have found the potential impact of mulch film (MF) thickness on the physical and chemical properties of soil, microbial communities, crop yields, etc. (Xiong et al., 2023). For instance, many soil physical and chemical properties, such as soil organic C, total nitrogen (TN), and soil bulk density (SBD), have significant differences because of various treatments of different film thicknesses, and the film thickness also significantly affects the residual rate of the film in the soil (Uzamurera et al., 2023). The changes in the soil environment further cause the alteration of soil metabolites and metabolic pathways (Wang Q. et al., 2023). However, research on the effects of BM thickness on the microbial metabolites of the rhizosphere soil is still lacking. More information is needed to determine whether and how mulch film thickness affects the composition of the rhizosphere soil microbial community and their associated metabolic function, especially the function of degrading exogenous pollutants and removing harmful molecules.

For this reason, in this study, based on high-throughput sequencing platforms, 16S rRNA sequencing was performed to reveal the changes in the abundance, diversity, and composition of the bacterial community in tobacco rhizosphere soil treated with different thicknesses of mulch films (MFs). The aim of this study was to (1) reveal the response of tobacco growth and yield to MF thickness, (2) explore the potential effects of MFs with various thicknesses on the physical and chemical properties of soil, and (3) determine the influence of the applications of different thickness MFs on the rhizosphere microbiome of tobacco plants. By delving into these aspects, this study provides a theoretical basis for the correlation between the soil microbial community and mulch thickness.

## 2 Materials and methods

### 2.1 Experimental site and design

The field experiment of this study was carried out from March to September 2023 in Shuangshu Village, Longjie Town, Chengjiang City, Yunnan Province, China (N 24°39′, E 102°52′). The elevation of the experimental area is ~1,741 m, and the soil type is laterite. The annual average temperature, extreme maximum temperature, and extreme minimum temperature are 17.2, 33.7 and −3.9°C, respectively. The average annual sunshine hours are 2,360.5 h, the annual average precipitation is 795.8 mm, the annual average relative humidity is 76%, the frost-free period is 318 days, and the dominant wind direction is southwest.

The tobacco variety used in this experiment was K326. Healthy tobacco seedlings with a height of 5–7 cm and having 4–5 leaves were prepared by floating germination and cultivation from 10 March 2023. Transplanting was carried out on 25 April 2023, with a tobacco planting density of ~16,500 plants per hectare (ha) and a plant spacing of 0.5 m × 1.2 m. The cultivation and management measures were carried out in accordance with local high-quality tobacco leaf production technology. The following fertilization standards were included: the N application rate (pure N) for flue-cured tobacco was 120 kg/ha, the P application rate (P_2_O_5_) was 90 kg/ha, and the potassium (K) application rate (K_2_O) was 321 kg/ha. The final number of leaves left per plant was 16–20, and the apical meristems were removed.

There were six treatments in this experiment: CK: non-mulching films (exposed soil), which comprised the control group; PET0.010 treatment: covering 0.010 mm polyethylene films; and BM0.004, BM0.006, BM0.008, and BM0.010 treatments: covering 0.004 mm, 0.006 mm, 0.008 mm, and 0.010 mm biodegradable films, respectively. Each treatment was replicated three times, with a total of 18 plots. Each plot was 12 m × 10 m (length × width), and the same agricultural management measures were adopted in all plots. The mulching films used in this experiment were all produced by Shanghai Hongrui Biotechnology Co., Ltd.

### 2.2 Investigation of agronomic characters

When the tobacco reached the technical maturity stage in September 2023, 15 tobacco plants were randomly selected to detect agronomic characters in each plot. Multiple agronomic traits, including tobacco plant height (TPH), stem girth (SG), leaf number (LN), and maximum leaf area (MLA), were investigated by referring to the methods of the national standard YC/T142-2010, “Investigating and measuring methods of agronomical character of tobacco.” The mature leaves were collected in three batches and baked using an intelligent baking method. The fresh leaf weight (FLW), and dry leaf weight (DLW) were the sum of the three batches before and after baking.

### 2.3 Soil sample collection

After the leaf harvest, the tobacco roots were dug out and the soil particle on the root surfaces was removed. Gentle brushing was used to remove and collect the rhizosphere soil still adhering to the roots; the collected soil from 10 tobacco plants was mixed as a composite sample in ziplock bags. Three composite samples were taken from each plot. One part was frozen in liquid nitrogen for soil DNA extraction, and the other part was placed in a cool, shaded area to air dry for soil property analysis.

### 2.4 Soil property analysis

The SWC and ST were measured using a soil parameter detector (Spectrum Technologies TDR350, USA). The SBD and soil porosity (SP) were determined by conducting the ring knife method. The soil pH was measured by a pH meter in a soil-water suspension (1:2.5). The SOM was determined according to the Yunnan local standard methods of NY/T1121.6-2006. The TN was determined by conducting the Kjeldahl method (Bremner, 1965). The available nitrogen (AN) levels, including ammonium, nitrate, and easily decomposable and hydrolyzable organic N, were determined using alkali distillation (Page et al., 1982). The total phosphorus (TP) and available phosphorus (AP) were extracted with HF-HNO_3_-HClO_4_ and sodium bicarbonate, respectively, and then determined by conducting the molybdenum-blue method (Page et al., 1982). The total K (TK) and available K (AK) were extracted with HF-HNO_3_-HClO_4_ and ammonium acetate, respectively, and then determined by flame photometry (Halajnia et al., 2009).

### 2.5 Soil DNA extraction and sequencing

Total genomic DNAs were extracted from 0.25 g of the rhizosphere soil per sample with a soil DNA extraction kit (DP336-02, Tiangen Biochemical Technology Co., Ltd., Beijing) following the manufacturer's protocol. The soil DNAs were quantified using a Nanodrop 2000 spectrophotometer (Thermo Scientific Inc., USA), and their integrity was detected by 1% agarose gel electrophoresis. Each treatment was repeated three times.

The bacterial 16S rRNA gene V3–V4 region of the sample was amplified using bacterial primers 341F (5′-ACTCCTACGGGAGGCAGCAG-3′) and 806R (5′-GGA CTACHVGGGTWTCTAAT-3′). The PCR reaction was as follows: 4 μl 5 × FastPfu Buffer, 2 μl dNTP (2 mmoL/L), 5 μmoL/L forward and reverse primers at 0.8 μl each, 2 μl DNA template, 0.4 μl 5 U/μl Taq polymerase, and 20 μl of ddH_2_O added. The reaction conditions were as follows: initial denaturation at 95°C for 3 min, followed by denaturation at 95°C for 30 s, annealing at 55°C for 30 s, and extension at 72°C for 30 s. This was repeated for 30 cycles and was followed by extension at 70°C for 5 min and preservation at 4°C.

The PCR amplification products from each sample replicate were mixed and purified using an AxyPrep DNA (Axygen Biosciences, Union City, CA, USA) gel recovery kit. The purified amplicon was quantified using a NanoDrop 2000 spectrophotometer (Thermo Scientific Inc., USA). The final sequencing library was sent to Beijing Biomarker Biotechnology Co., Ltd. for high-throughput soil bacterial 16S rRNA sequencing on the PacBio platform.

### 2.6 Sequencing data analysis

To ensure the quality and reliability of the sequencing data, a series of quality control steps were performed. Lima (version 1.7.0) software was used to identify the CCS file obtained by sequencing and obtain the Raw-CCS sequence. Cutadapt (version 1.9.1) software was used to identify and remove primer sequences from the sequences, which resulted in a Clean-CCS sequence devoid of any primer residues; this was followed by chimera removal using UCHIME (version 4.2). Finally, an Effective-CCS sequence was obtained for subsequent analysis.

USEARCH software was used to cluster reads at the similarity level of 97.0% and obtain operational taxonomic units (OTUs) (Edgar, 2013). Taxonomy annotation of the OTUs was performed based on the Naive Bayes classifier in QIIME (Bolyen et al., 2019) using the Silva database (Quast et al., 2013).

The α-diversity measures were calculated and displayed using QIIME software for diversity analysis, and KRONA software was used for visualization. The microbial community α-diversity was evaluated using the Ace, Chao1, Shannon, and Simpson indices. β-diversity was determined to evaluate the degree of similarity of the microbial communities from different samples using QIIME. Principal coordinate analysis (PCoA) and non-metric multidimensional scaling (NMDS) were used to analyze the β-diversity. The PCoA based on the Bray–Curtis distance was applied to analyze the species diversity differences among multiple samples. NMDS (Looft et al., 2012) is used to reduce the research objects in multidimensional space to low-dimensional space for localization, analysis, and classification while preserving the original relationship between the objects. Furthermore, we employed the linear discriminant analysis (LDA) effect size (LEfSe) (Segata et al., 2011) to test the significant taxonomic difference among the groups. A logarithmic LDA score of 4.0 was set as the threshold for discriminative features. To investigate the differences in the microorganism composition among various factors, a redundancy analysis (RDA) was conducted using the “vegan” package in R. Gtree Extra to obtain the evolutionary relationship between the species and the relative abundance ratio of the species among different soil samples.

Gene function differences were analyzed using the Kyoto Encyclopedia of Genes and Genomes (KEGG) database. PICRUSt2 software was used to annotate species between the feature sequences to be predicted and the existing phylogenetic tree in the software, and IMG microbial genome data were used to output functional information and then infer the functional gene composition in the samples (Parks et al., 2014). The composition and differences of the metabolic pathways were then analyzed using the KEGG database.

### 2.7 Statistical analysis

The tobacco agronomic characteristics, physical and chemical properties of soil (PCPS), and soil microorganism differences were statistically analyzed using one-way analysis of variance (ANOVA) with the Tukey *post hoc* test to determine whether the concentration levels were significantly different between the control and treatments. IBM SPSS (version 26) software was used for statistical analysis, and GraphPad Prism (version 8.0) software was used to generate charts.

## 3 Results

### 3.1 Effect of MFs on tobacco growth

Overall, there was a significant positive correlation between the multiple agronomic traits such as TPH, SG, LN, MLA, FLW, and DLW and the MF thickness (Supplementary Table 1). In addition, the average TPH, LN, and FLW of all MF treatments were 4.46%, 7.14%, and 4.61% higher, respectively, compared with those without MFs (Supplementary Figure 1). In addition, the agronomic properties of the tobacco-mulched PET0.010 films had no significant differences with those of BM0.010 mm. However, the yield traits in the field under BM0.004 mm were significantly lower compared with those under other types of MFs (Table 1).

**Table 1:** Comparison of the main agronomic traits of the tobacco under different treatments.

	**CK**	**BM0.004**	**BM0.006**	**BM0.008**	**BM0.010**	**PET0.010**
TPH (cm)	112.00 ± 1.87 b	115.60 ± 2.41 ab	116.40 ± 2.30 ab	117.60 ± 2.88 a	118.20 ± 2.39 a	117.20 ± 2.39 a
SG (cm)	11.68 ± 0.16 a	11.66 ± 0.19 a	11.86 ± 0.18 a	11.76 ± 0.15 a	11.92 ± 0.23 a	11.96 ± 0.18 a
LN	16.80 ± 0.83 b	16.60 ± 0.89 b	17.80 ± 1.10 ab	18.00 ± 0.71 ab	18.60 ± 1.14 a	19.00 ± 0.71a
MLA (cm^2^)	768.00 ± 12.31 a	776.80 ± 8.35 a	782.80 ± 7.05 a	785.00 ± 7.42 a	784.60 ± 8.08 a	785.20 ± 11.56 a
FLW (kg)	36.86 ± 0.83 b	37.73 ± 0.77 ab	38.68 ± 0.91 ab	38.00 ± 0.89 ab	39.37 ± 1.05 a	39.00 ± 1.22 a
DLW (kg)	6.65 ± 0.08 c	6.93 ± 0.21 bc	7.18 ± 0.07 ab	7.27 ± 0.17 ab	7.45 ± 0.18 a	7.35 ± 0.30 a

Data are provided as means ± SDs; different lowercase letters in the same row indicate significant differences (*p* < 0.05).

**Figure 1:**
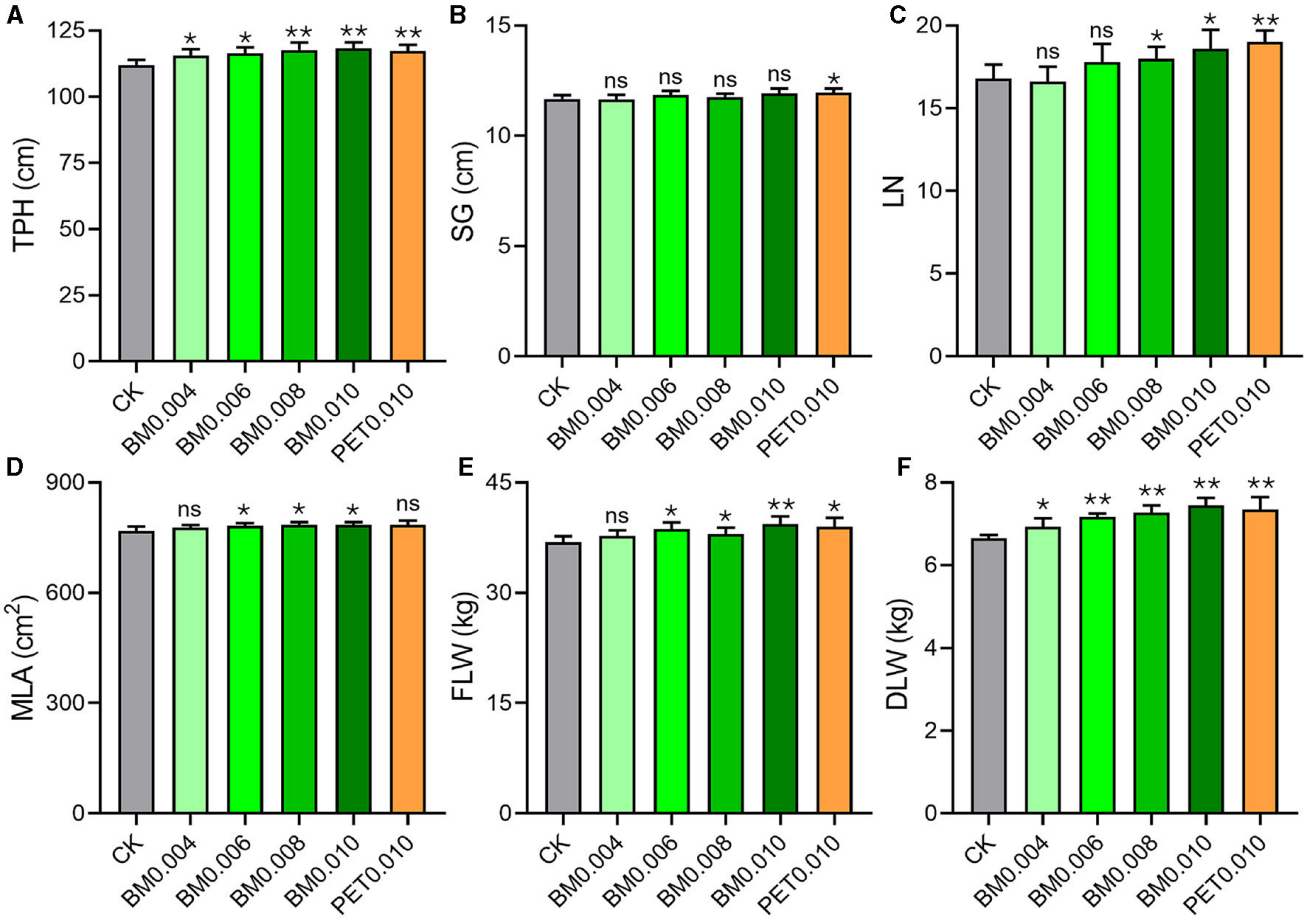
Effects of different MF treatments on the agronomic characteristics of the tobacco plants. **(A)** TPH, tobacco plant height. **(B)** SG, stem girth. **(C)** LN, leaf number. **(D)** MLA, maximum leaf area. **(E)** FLW, fresh leaf weight. **(F)** DLW, dry leaf weight. CK, control group (non-mulching). Error bars represent the standard deviations; ns indicates *p* > 0.05, * indicates *p* < 0.05, and ** indicates *p* < 0.01 compared to the CK.

As shown in Figure 1A, the TPH was significantly higher (*p* < 0.05) in the BM0.004 and BM0.006 groups, and it was extremely significantly higher (*p* < 0.01) in the PET0.010, BM0.008, and BM0.006 groups than in the control group. Among the five MF groups, there were no significant differences in the SG between the BM groups and the control group, but the SG of the PET0.010 group was significantly higher than that of the control group (Figure 1B). With the increase of the film thickness, the LN in the BM0.008, BM0.010, and PET0.010 groups began to show a noticeable increase compared with that in the control group, and the LN in the PET0.010 group showed the most increase (Figure 1C). The treatment of MFs also affected the MLA and FLW of the tobacco plants. When the MF thickness was >0.004 mm, the MLA and FLW of the BM treatment were significantly higher (*p* < 0.05) than those of the control, and the MLAs of BM0.006, BM0.008, and BM0.010 were 1.93%, 2.21%, and 2.16% higher than those of the control group, respectively (Figure 1D). The FLAs in the M0.006, BM0.008, BM0.010, and PET0.010 groups were 4.95%, 3.09%, 6.81%, and 5.82% higher than those in the control group, respectively (Figure 1E). More importantly, the thickness of the MFs significantly affected the tobacco DLW. Compared with the control group, the increased ratios of the DLW were 4.12%, 7.91%, 9.29%, 12.00%, and 10.52% in the BM0.004, BM0.006, BM0.008, BM0.010, and PET0.010 groups (Figure 1F). The higher DLW of 0.58 ± 0.28 kg occurred with the MFs, and it was ~8.72% of that without the MFs (Supplementary Figure 1F).

### 3.2 Influence of mulch films on the soil properties

**Figure 2:**
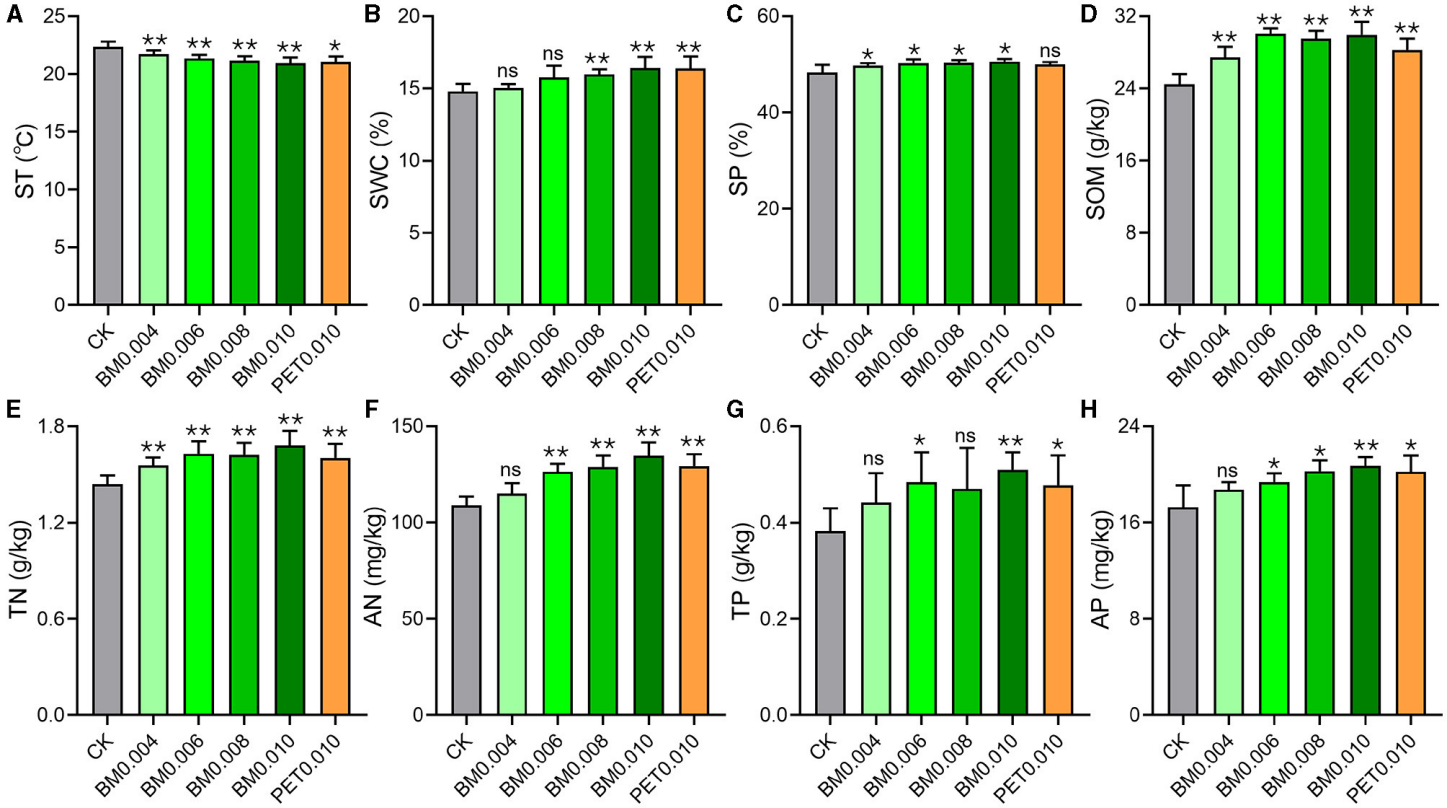
Effects of different MF treatments on the soil properties. **(A)** ST, soil temperature. **(B)** SOM, soil organic matter. **(C)** SP, soil porosity. **(D)** SWC, soil water content. **(E)** AN, available nitrogen. **(F)** AP, available phosphorus. **(G)** TN, total nitrogen. **(H)** TP, total phosphorus. CK, control group (non-mulching). Error bars represent the standard deviations; ns indicates *p* > 0.05, * indicates *p* < 0.05, and ** indicates *p* < 0.01 compared to the CK.

We evaluated multiple PCPS in the field with the different thicknesses and types of MFs and the control group (Figure 2). There were no significant differences observed in the SBD, pH, AK, and TK between the MFs groups and the control group (Supplementary Figure 2). In addition, the correlation analysis results indicated that multiple PCPS, such as SOM, SP, SWC, TN, AN, TP, and AP, showed a positive correlation with the MF thickness, while the ST showed a negative correlation with the MF thickness (Supplementary Table 1).

As shown in Figure 2A, with the increase in the MF thickness, the ST was found to have a downward trend, from 22.35°C in the CK to 20.93°C in the BM0.010. However, the other indices of the PCPS improved with the increase in the MF thickness (Figures 2B–H). Especially, the SOM and TN evidently increased (*p* < 0.01) once the soil was mulched with the MF (Figures 2D, E). The AN, TP, and AP of the soil began to have significant differences when the MF thickness was >0.004 mm (Figures 2F–H). In the soil mulched with the MF thickness >0.06 mm, the SWC appeared to be significantly different from that in the control group (Figure 2B). Interestingly, except for the PET0.001 group, there were obvious differences (*p* < 0.05) in the SP between all BM groups and the control group (Figure 2C).

### 3.3 Response of the rhizosphere soil microbial community to residual mulch films

#### 3.3.1 Effects of MFs on the diversity of the rhizosphere soil bacterial community

**Figure 3:**
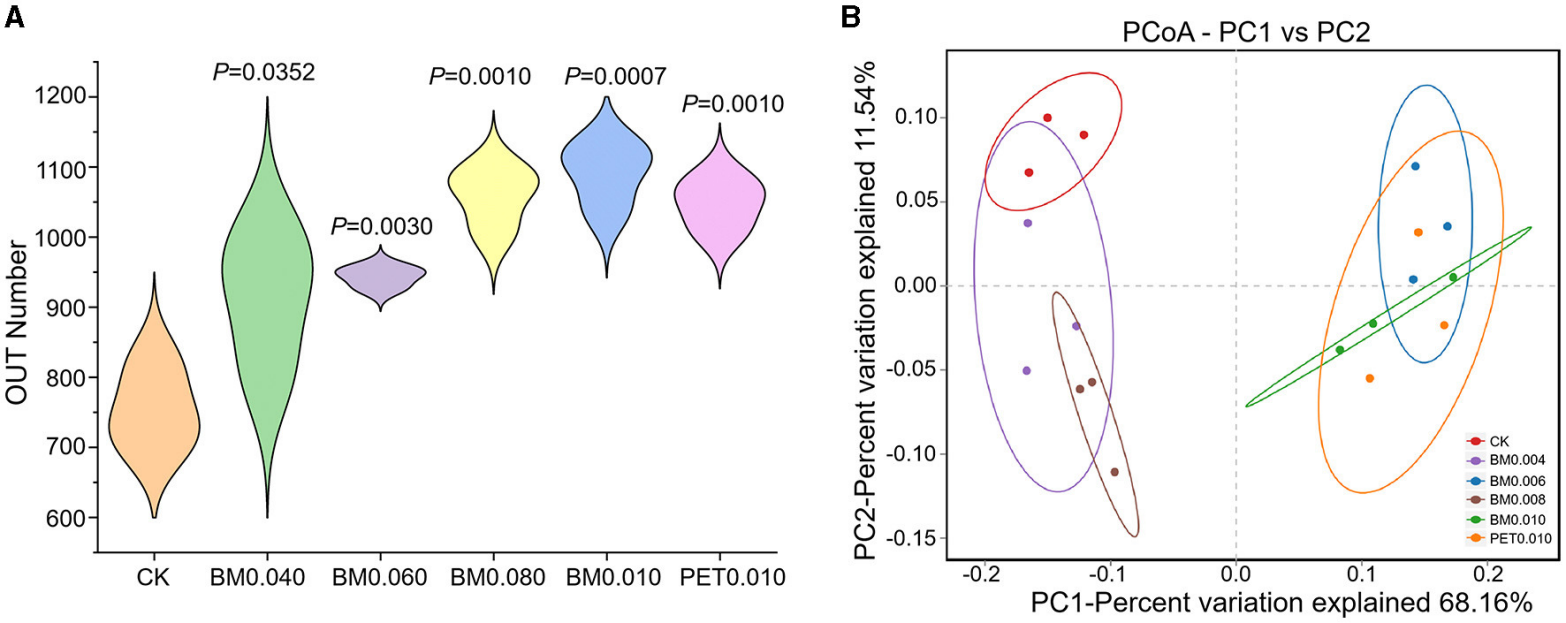
The differences in the soil microbial diversity under different treatments. **(A)** The differences in the soil microbial diversity under different treatments. **(B)** Principal coordinate analysis (PCoA) of the soil bacteria under different treatments.

To further clarify the response of the rhizosphere soil microorganisms to the MFs in the Yunnan laterite, 18 soil samples treated with and without the mulch films were subjected to high-throughput soil bacterial 16S rRNA sequencing. A total of 231,437 high-quality reads of bacteria remained in the dataset, with an average of 1,446 bp. Through clustering operations, the optimized sequences were classified into operational taxonomic units (OTUs) according to their similarity. With a 3% dissimilarity threshold, the sequences were classified into 1,976 OTUs in the bacterial communities using the Ribosomal Database Project (RDP) classifier. The violin plot showed that the soils under the different types of MFs exhibited a greater number of OTUs than the control soils (Figure 3A). The number of OTUs in the soil treated with BM0.006, BM0.008, BM0.010, and PET0.10 was significantly greater than that in the control group (*p* < 0.01), and the maximum number of the OUTs was 1,091 in the BM0.010 group (Figure 3A).

To quantify the diversity and richness of the microbial community of the rhizosphere soils between the control and MF treatments, the α-diversity of the microbial community was evaluated using the Ace, Chao1, Shannon, and Simpson indices within a single microbial ecosystem, as shown in Table 2. The PD whole tree indices of the mulched soil were larger than those of the control soil, suggesting that the microbial diversity in the mulched soil was higher. The coverage indices from the 18 rhizosphere soil samples were >97.00%, showing that the sequencing capacity was acceptable. In the bacterial communities, the richness (Ace and Chao1 indices) and diversity (Shannon and PD whole tree indices) of the mulch group were higher compared to the control group. Among them, the richness and diversity of the BM0.010 mm mulch group were the highest, and the diversity (PD whole tree indices) of the BM0.008 mulch group was the highest. The results suggested that the mulch treatment affected the bacterial communities and that the thicknesses and types of MFs had different effects on the rhizosphere soil bacterial community.

**Table 2:** The diversity and richness indices of the soil bacterial communities.

	**Ace**	**Chao 1**	**Shannon**	**PD_whole_tree**	**Simpson (%)**	**Coverage (%)**
CK	922.91 ± 101.76 b	907.33 ± 114.34 b	7.60 ± 0.08 c	46.47 ± 3.86 c	97.91 ± 0.42 b	97.88 ± 0.44 a
BM0.004	1,095.47 ± 114.58 ab	1,121.13 ± 123.31 ab	8.22 ± 0.32 b	55.38 ± 4.38 ab	99.11 ± 0.31 a	97.58 ± 0.30 a
BM0.006	1,109.82 ± 35.56 ab	1,130.48 ± 56.22 ab	8.46 ± 0.03 ab	54.01 ± 0.71 bc	99.27 ± 0.09 a	97.36 ± 0.41 a
BM0.008	1,249.08 ± 45.75 a	1,251.28 ± 41.46 a	8.71 ± 0.09 a	62.40 ± 1.56 a	99.41 ± 0.07 a	97.20 ± 0.08 a
BM0.010	1,303.47 ± 80.27 a	1,306.81 ± 82.74 a	8.74 ± 0.07 a	61.26 ± 2.75 ab	99.46 ± 0.02 a	97.88 ± 0.39 a
PET0.010	1,251.43 ± 65.25 a	1,255.38 ± 89.46 a	8.63 ± 0.19 ab	60.37 ± 2.94 ab	99.37 ± 0.16 a	97.04 ± 0.46 a

Data are provided as means ± SDs; different lowercase letters in the same column indicate significant differences (*p* < 0.05).

To get a better insight into the differences of the rhizosphere soil microbial communities, PCoA was applied to evaluate the microbial community β-diversity. As shown in Figure 3B, the samples of the control group and mulch group were distributed separately at 68.16 and 11.54% on the PCoA vector x and y axes for the bacterial community. The rhizosphere soil samples of the control group, BM0.004, and BM0.008 were distinct from those of the BM0.006, BM0.010, and PET0.010 mulch group, demonstrating that large microbial community differences were affected by the thicknesses and types of MFs.

#### 3.3.2 Effects of MFs on the structure of the rhizosphere soil bacterial community

**Figure 4:**
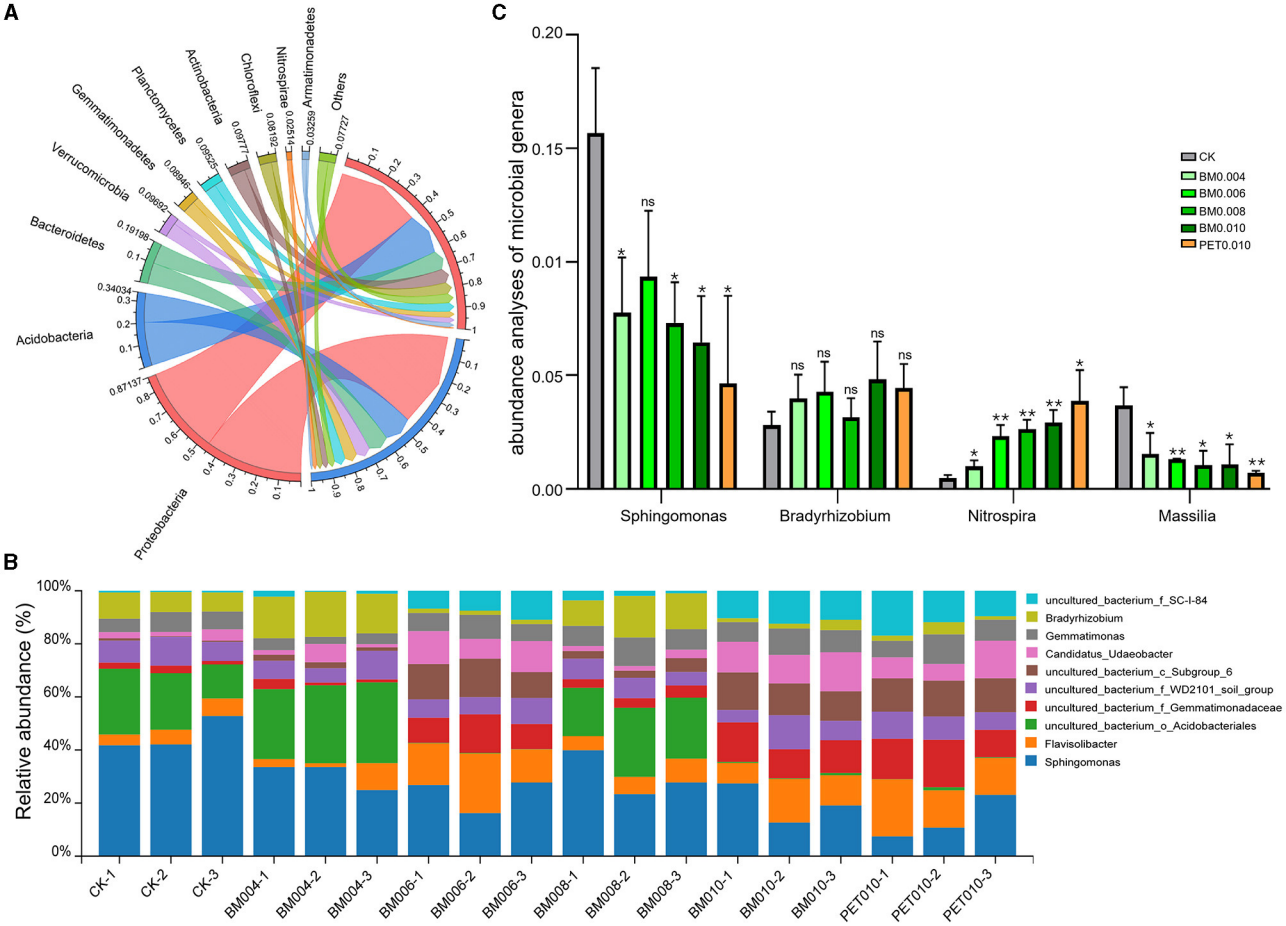
Composition and abundance of the soil microorganisms in the MF-treated and control samples. **(A)** Composition of the bacteria at the phylum level. **(B)** Abundance of the bacteria at the genus level. **(C)** Comparison of the differences among the microbial genera. Error bars represent the standard deviations; ns indicates *p* > 0.05, * indicates *p* < 0.05, and ** indicates *p* < 0.01 compared to the CK.

Circos graphs and bar plots show the microbial community composition and abundance at the phylum and genus levels of the bacteria in the exposed and mulch film soils (Figures 4A, B). The bacterial phyla with a high abundance in the rhizosphere soil samples were *Proteobacteria, Actinobacteria, Bacteroidetes, Verrucomicrobia, and Gemmatimonadetes*, accounting for 45.14%, 14.84%, 9.83%, 6.26%, and 5.47%, respectively. The bacterial genera with the highest abundance were *Sphingomonas* and *Flavisolibacter*, accounting for 8.54% and 3.37%, respectively. It is worth noting that, compared with the control group, the mulch treatment had apparent impacts on the composition and abundance of the main soil bacterial phyla and genera. For example, among the top 10 most abundant phyla, six phyla, including *Proteobacteria*, increased, while four phyla, including *Actinobacteria*, decreased in the mulched soil; among the top 10 most abundant genera, six genera, including *Flavisolibacter* and *Gemmatimonas*, increased (Figure 4A), while four genera, including *Sphingomonas* and *Bradyrhizobium*, decreased (Figure 4B).

In addition, *Sphingomonas, Bradyrhizobium, Nitrospira*, and *Massilia* were closely related to the changes of the MF thickness at the microbial genus level. There was a significant positive correlation between *Bradyrhizobium, Nitrospira*, and the MF thickness, and there was a significant negative correlation between *Sphingomonas, Massilia*, and the MF thickness (Supplementary Table 1). Compared with the control, the abundances of *Sphingomonas* significantly decreased, 50.52%, 40.43%, 53.35%, 58.83%, and 70.44%, and the abundances of *Massilia* decreased averagely, 58.04%, 64.73%, 71.68%, 70.30%, and 80.51% in the BM0.004, BM0.006, BM0.008, BM0.010, and PET0.010 treatment, respectively (Figure 4C). With the increase of the film thickness, the abundances of *Bradyrhizobium* in the BM0.004, BM0.006, BM0.008, BM0.010, and PET0.010 groups were 1.42, 1.52, 1.12, 1.71, and 1.58 times, and the abundances of *Nitrospira* were 2.13, 5.04, 5.73, 6.33, and 8.39 times those of the control group (Figure 4C).

#### 3.3.3 Mulch thickness sensitive bacterial populations

**Figure 5:**
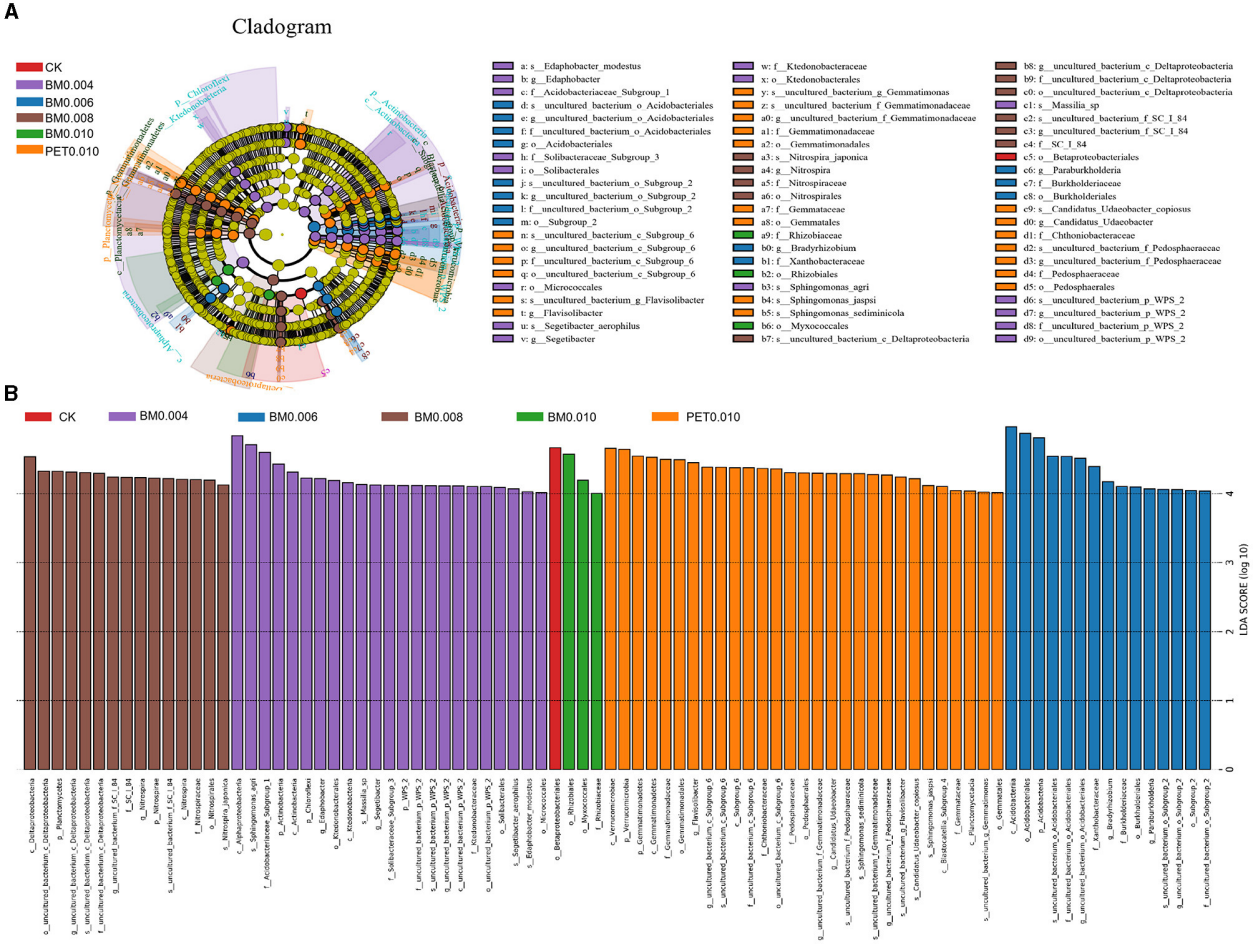
LEfSe results for the control and various types of mulch films. **(A)** LEfSe cladogram. **(B)** LEfSe bar chart.

To determine the classified bacterial taxa with significant abundance differences between the various thicknesses of the MF treatment and control soil, a biomarker analysis using the LDA method was performed (Figure 5, Supplementary Table 2). The mulch film treatment promoted the reproduction of *Proteobacteria, Verrucomicrobia, Gammaproteobacteria, Deltaproteobacteria*, and other bacterial orders and of the families of *Betaproteobacteriales, Pedosphaeraceae, Myxococcales, Xanthomonadales*, and *Pedosphaerales*. In addition, it inhibited the reproduction of the bacteria such as the families of *Sphingomonadaceae* and *Burkholderiaceae*, as well as the genera of *Sphingomonas*. The upward or downward trends of the bacteria reproduction were closely related to the thickness of the MFs, but there was no significant difference between the different types of MFs at the same film thickness.

#### 3.3.4 Alteration in the soil microbial function

**Figure 6:**
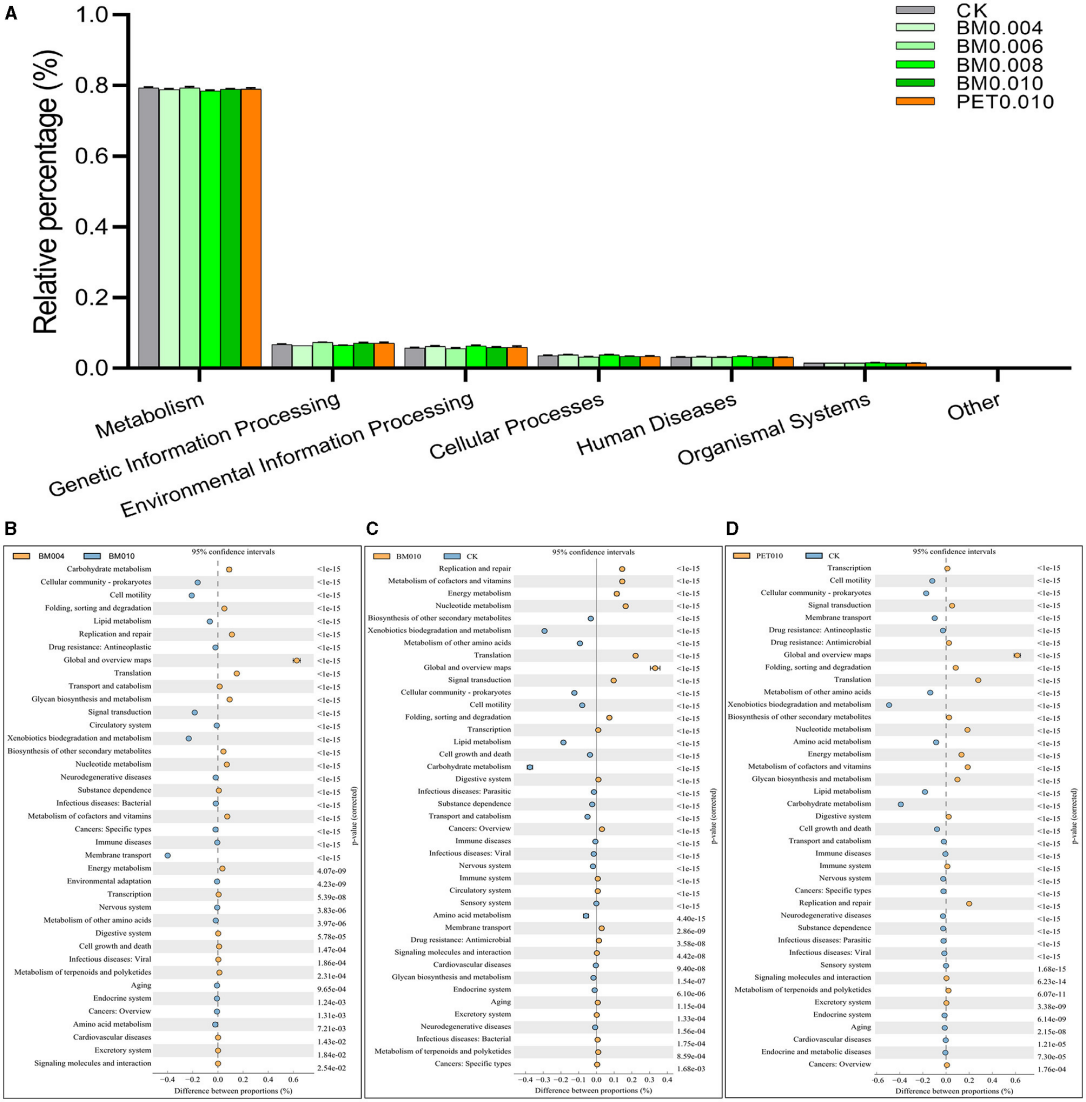
The changes of the predicted functional genes in different metabolism pathways by the KEGG. **(A)** Relative percentages at the L1 level. **(B)** BM0.040 vs. BM0.010. **(C)** BM0.010 vs. CK. **(D)** PET0.010 vs. CK.

Alteration in the microbial community may further lead to changes in the overall metabolic functions of the soil microbial community, including changes in the ability to degrade exogenous pollutants. To this end, the test results were analyzed based on the 16S KEGG PICRUSt function prediction. All predicted functional genes in the KEGG L1 classification level mainly included (Control: BM0.004: BM0.006: BM0.008: BM0.010: PET0.010): Metabolism (79.34%: 78.86%: 79.34%: 78.46%: 78.96%: 79.02%), genetic information processing (6.72%: 6.42%: 7.32%: 6.47%: 7.10%: 7.14%), environmental information processing (5.76%: 6.20%: 5.60%: 6.37%: 5.91%: 5.93%), cellular processes (3.56%: 3.80%: 3.16%: 3.83%: 3.37%: 3.34%), human diseases (3.13%: 3.21%: 3.10%: 3.33%: 3.15%: 3.10%), and organismal systems (1.49%: 1.51%: 1.47%: 1.54%: 1.50%: 1.48%; Figure 6A). The microbial function at the KEGG L1 level changed in the rhizosphere soil due to the different thicknesses and types of mulch films, but it was not significant.

More detailed function changes that occurred after the mulch film treatment were refined through the calculation of expression abundance multiples (Figures 6B–D). It was observed that the metabolic function category, the metabolic processes such as the metabolism of cofactors and vitamins, nucleotide metabolism, and energy metabolism were higher in the soil mulched BM0.010, while the functions such as carbohydrate metabolism, xenobiotics biodegradation and metabolism, and lipid metabolism were higher in the control group (Figure 6B). The results were similar to those of the microbial function changes between the PET0.010 group and the control group (Figure 6D). In addition, the results also showed that the xenobiotics biodegradation and metabolism, membrane transport, and cell motility were up-regulated, while the carbohydrate metabolism and translation were down-regulated with the increase of the mulch thickness (Figure 6C).

### 3.4 Relationship between the PCPS and the microbial communities

**Figure 7:**
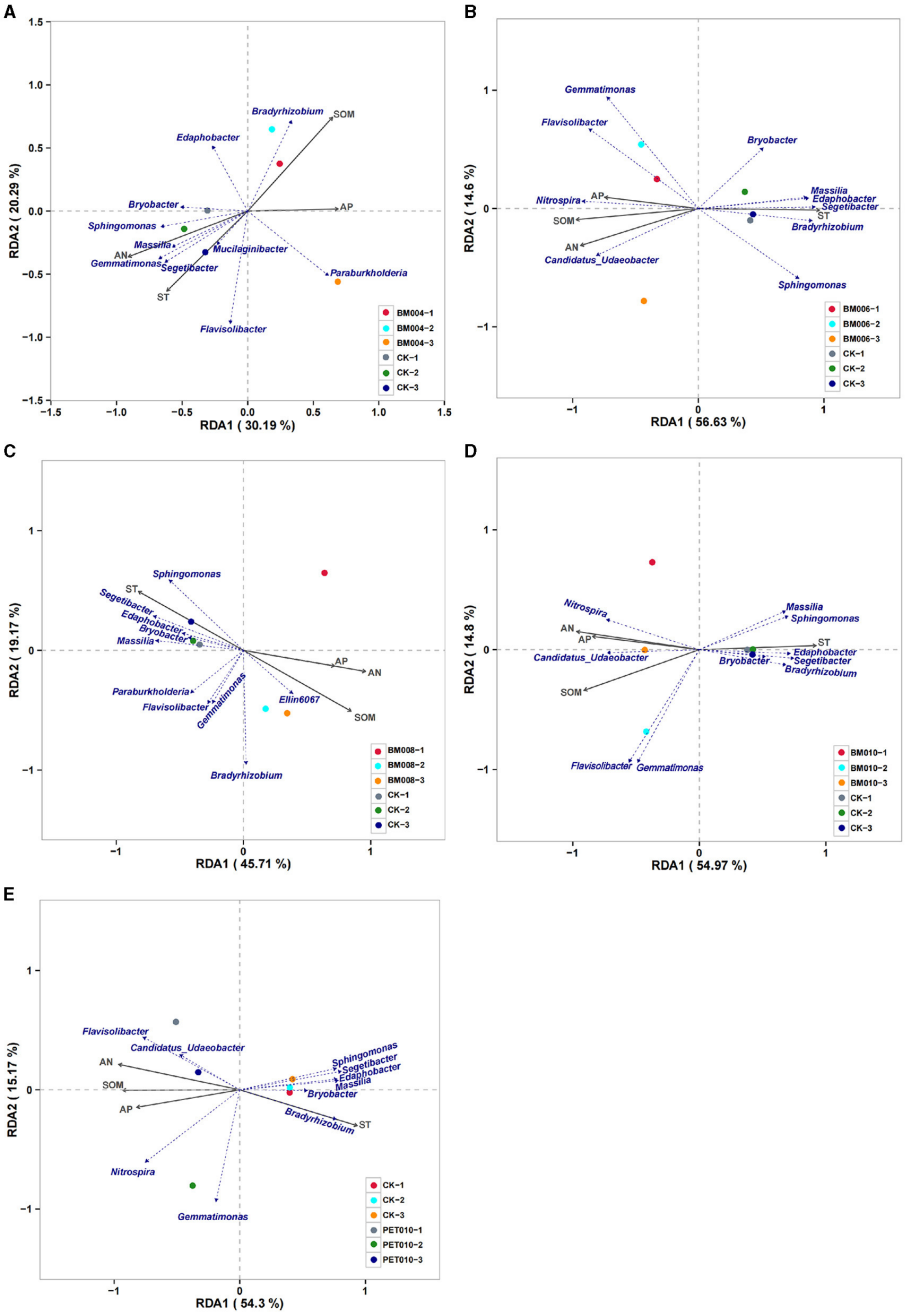
The redundancy analysis (RDA) of the PCPS and microbial communities' genus levels of different thicknesses and types of mulch management treatments in the tobacco field. **(A)** BM004 vs. CK. **(B)** BM006 vs. CK. **(C)** BM008 vs. CK. **(D)** BM010 vs. CK. **(E)** PET010 vs. CK. ST, soil temperature; SOM, soil organic matter; AN, available nitrogen; AP, available phosphorus.

The RDA summarized the connections between the PCPS of the different thicknesses and types of mulch films and the genus of the microbial communities (Figure 7). The correlation between the percentages of the SOM, AN, and AP was positive, while the correlation between the percentages of the SOM, AN, AP, and ST was negative for all mulch films.

The PCPS and the composition of the microbial communities had obvious correlations in different aggregates. In addition, the results showed that the ST, SOM, and AN of the rhizosphere soil had a greater influence on the changes of the microbial communities than the AP. Regarding the RDA, RDA1 explained 30.19%−56.63% and RDA2 explained 14.60%−20.29%. The combined interpretation of the two axes was 50.48%−71.23% of the microbial community changes in the various mulches. This interpretation showed that the PCPS in the soil treated with various thickness mulches showed a good degree of explanation for the changes in the microbial community.

Specifically, *Sphingomonas, Bryobacter, Massilia*, and *Segetibacter* were closely related to changes of the ST; *Bradyrhizobium* and *Nitospira* were closely related to the changes of the SOM, AN, and AP in the soil. Among them, there was a significant positive correlation between *Sphingomonas, Bryobacter, Massilia*, and the ST in different aggregates. *Bradyrhizobium* was significantly negatively correlated with the SOM, AN, and AP, while *Nitospira* was significantly positively correlated with the SOM, AN, and AP. In addition, *Flavisolibacter* was less related. *Bryobacter* had a positive correlation with *Sphingomonas* and *Segetibacter* but was negatively correlated with *Nitospira*. However, *Flavisolibacter* was less related.

## 4 Discussion

### 4.1 Influence of mulch films on the agronomic traits and yield of tobacco plants

Mulch films including biodegradable and PET mulches have been widely adopted in agricultural cultivation for a long time. The general impact of mulches on the economy and environment has been fully recognized (Gan et al., 2013; Liu et al., 2014). For instance, a mulching film (use of soil covers, such as plastic or organic) is a widely used cropping approach to enhance the growth and yield of crops. Mulches are applied to the soil surface for the retention of soil moisture and SOM, the regulation of ST, and the suppression of weed growth (Li et al., 2013). Mulching also has a positive effect on crops with an improved lettuce head, leaf and stem growth, and total yields compared to bare ground (Verdial et al., 2001; Xu et al., 2016). Similarly, there were significant phenotype changes of the tobacco including the TPH, LN, FLW, and DLW between the mulching films and the control groups (Supplementary Figure 1). The most striking characteristic of the tobacco plants covered by the film was the biomass increase. In addition, the agronomic traits of the tobacco were better under the mulching treatment with increased thickness (Figure 1). In addition, the Mantel test results showed a significantly positive correlation between the TPH, LN, FLW, and DLW and the thickness of the MFs (Supplementary Table 1). The tobacco yield increase may have been caused by an improvement in the content of the soil water and nutrients as the film thickened. The relevant findings of this study further corroborated the conclusions drawn from related research on the use of mulches, where it was observed that the use of mulches may promote plant growth and increase crop yield (Dong et al., 2009, 2018).

### 4.2 Influence of mulch films on the physical and chemical properties of soil

Numerous studies have highlighted the obvious effects of MFs on the physical and chemical properties of agricultural soil as soil C, N, and phosphorus cycles (Monaco et al., 2008; Zhao et al., 2013). Simultaneously, the polyester fibers of MFs applied using the planting method can significantly affect the water-holding capacity of soil (Ali et al., 2016). Consistent with these findings, the results of this study indicated that the SBD, SWC, and AP of the soil mulched with the MFs were significantly increased compared with the exposed soil (*p* < 0.05). Furthermore, the increase of the ST, SOM, TN, and AN of the rhizosphere soil mulched with the MFs reached a very significant level (*p* < 0.01; Supplementary Figure 3). However, the SBD, pH, TK, and AK did not change depending on whether the soil was mulched or not (Supplementary Figure 2). The results of the current study also showed that the PCPS was significantly positively correlated with the thickness of the MFs (Supplementary Table 1). This may be due to the MFs influencing the water, N, and P content and P transformation processes by stimulating enzyme activity and altering the composition of microbial communities (Li et al., 2014; Sintim et al., 2019; Ding et al., 2021; Zhang et al., 2024a).

In addition, the correlation analysis results showed that the strongest correlation was between the MF thickness and soil AN (Supplementary Table 1). When the thickness of the BM was 0.010 mm, the soil AN increased by 24.94% compared to CK (Figure 2F). An increasing number of studies have shown a strong correlation between the microbial community structure and soil N content (Kelly et al., 2022). In agricultural ecosystems, soil bacteria and fungi play a crucial role in regulating N transformation. Fungal communities are crucial functional contributors to the breakdown of organic matter and N cycling (Marí et al., 2020). Research has shown that film mulching affects the abundance of soil microbes by increasing ST and nutrients, thereby altering microbial N-cycling genes and processes (Zhou et al., 2021). However, in this study, the ST decreased with the increase of the film thickness, which may be because the soil was measured at a higher air temperature. In addition, mulching films can regulate the ST for promoting the growth of plants. However, further research is needed for determining how the N-cycling mechanism responds to the combined effects of rhizosphere microbes and MF thickness.

### 4.3 Influence of mulch films on the shaping of the rhizosphere microbiota

The rapid acidification rate and nutrient loss of the krasnozem soil are the main factors reducing microbial abundance and restricting crop growth (Shen et al., 2021). By using the metagenomic analysis strategies, the richness and diversities of the tobacco rhizosphere microbiota in the Yunnan laterite were identified and found to have significant differences in the alpha diversity index based on the OTU (Figure 3A), Ace, Chao1, Shannon, PD whole tree, and Simpson indices (Table 2) between the mulching group and the control group, indicating significant differences in the rhizosphere soil microbial abundance and the diversity among the various soil treated with the different types and thicknesses of MFs, which suggests that mulching may promote the abundance and diversity of the rhizosphere microbial in the Yunnan laterite. As suggested by a previous study, mulching treatments as materials for modifying soil likely causes decreases in evaporation at the soil surface and reduces the rates of leaching, both contributing toward increasing soil moisture and nutrient retention (Zhang et al., 2012, 2017; Dou et al., 2023), which are favorable factors for soil microbial growth. In this study, the significantly higher diversity of the soil microbial growth was found under the thicker mulch treatment, while there was no significant difference between the two types of MFs, BM and PET (Table 2). It suggests that different types of MFs with the same thickness have little difference in their soil improvement ability.

Further key taxa in the genera level of the microorganisms related to the membrane thickness and PCPS were selected by conducting an RDA, and the abundance analyses of the key genera showed that *Bradyrhizobium* and *Nitrospira* (*p* < 0.05) were higher in the mulching groups (Figure 4C), while *Sphingomonas* and *Massilia* were significantly (*p* < 0.05) lower in the mulching groups (Figure 4C). The *Sphingomonas* genus suggested in current literature offers more extensive benefits, such as environmental bioremediation of heavy metals (Silver and Phung, 1996; Khan et al., 2014), biodegradation of polycyclic aromatic hydrocarbon (PAH) (Chen et al., 2008), and promotion of plant growth under different abiotic stress conditions, such as drought, salinity, and heavy metal stresses (Yang et al., 2014; Halo et al., 2015; Ali et al., 2019). The significant decrease of the abundance of *Sphingomonas* in the mulching group may be related to the reduction of the soil temperature, which resulted from long-term mulching, indicating that the degradation capacity of the mulching soil will decrease over time. *Massilia* was efficient in synthesizing multiple secondary metabolites and enzymes, degrading PAH and phenanthrene, and resisting heavy metals during previous studies (Turnbull et al., 2012; Feng et al., 2016; Lou et al., 2016; Shaffer et al., 2023). The increase of the film thickness led to the abundance decrease of *Massilia*, further indicating that mulching may reduce the biodegradability of soil. *Bradyrhizobium*, inhabiting the root nodules of legumes, may exhibit plant growth-promoting properties and may enhance the efficiency of nodulation and N-fixation (Michel et al., 2020; Thiago et al., 2016). In this study, the enrichment degree of *Bradyrhizobium* was higher with the increase of the film thickness, suggesting that all mulching treatments could increase the soil N, P, and SOM, which positively affects the growth of *Bradyrhizobium* and tobacco plants.

Moreover, numerous investigations have recognized that *Nitrospira* is a key and predominant nitrite-oxidizing bacteria (NOB) in biological nutrient removal systems, especially under low dissolved oxygen and substrate conditions (Mehrani et al., 2020; Hu et al., 2021). *Nitrospira* plays a major role in the environment; therefore, current research on NOB also mainly focuses on *Nitrospira* (Kuypers et al., 2018). Studies have found that *Nitrospira* can not only oxidize nitrite but also urea, ammonia, and cyanate to obtain energy, which shows that it has metabolic diversity in other pathways of the N-cycle (Daims et al., 2015). *Nitrospira* is responsible for various environmental factors that are sensitive; thus, different environmental factors also significantly affect the niche differentiation of NOB (Sun et al., 2021). In addition, the increase of MF thickness may lead to a decrease in soil dissolved oxygen, which is the reason for the abundance increase of *Nitrospira*. For tobacco growth, the enrichment of *Nitrospira* can also help catalyze the harmful molecules in the soil, such as nitrite oxidation. In this study, as the MF thickened, the abundance of *Nitrospira* also increased. This was also one of the reasons for the increased tobacco yield under the mulching film.

## 5 Conclusion

The results of this study showed that the utilization of different thicknesses of MFs significantly altered the agronomic characters and yield of tobacco, PCPS, and microorganism community abundance. There was a noticeable rise in the TPH, LN, and FLW of the tobacco treated with the MFs compared with the control group. Furthermore, these agronomic properties gradually improved with the increase of the film thickness but had little correlation with the type of MF. In the mulched soil, multiple PCPS, such as the SP, ST, SOM, SWC, TN, AN, TP, and AP, were closely related to the changes of the film thickness. Except for the ST, other PCPS were positively correlated with the thickness of the film. As the film got thicker, the contents of AN and AP increased the fastest, which provided an advantageous soil environment for the accumulation of the biological yield of tobacco. In addition, the MF also significantly changed the abundance, structure, and diversity of the tobacco rhizosphere microorganism community and interfered with the symbiosis networks of the bacterial community. The application of the mulch film favored the microorganisms' enrichment of *Bradyrhizobium* and *Nitrospira*, while the growth of *Sphingomonas* and *Massilia* was inhibited. Hence, our findings indicate that the utilization of soil mulching technology might lead to a reduction in the degradation function of microbial communities but might lead to an enhancement in the N utilization and the removal of hazardous molecules in the tobacco rhizosphere soil in the Yunnan laterite.

## Data Availability

The datasets presented in this study can be found in online repositories. The names of the repository/repositories and accession number(s) can be found in the article/Supplementary material.
