# Supplementary material for: Effects of mulch films with different thicknesses on the microbial community of tobacco rhizosphere soil in Yunnan laterite
Source: Front Microbiol. 2024 Sep 23;15:1458470. doi: 10.3389/fmicb.2024.1458470 (PMC11456438; doi:10.3389/fmicb.2024.1458470)
Supplement: Supplementary file 1 [file Data_Sheet_1.zip › Supplementary figures, tables.docx]

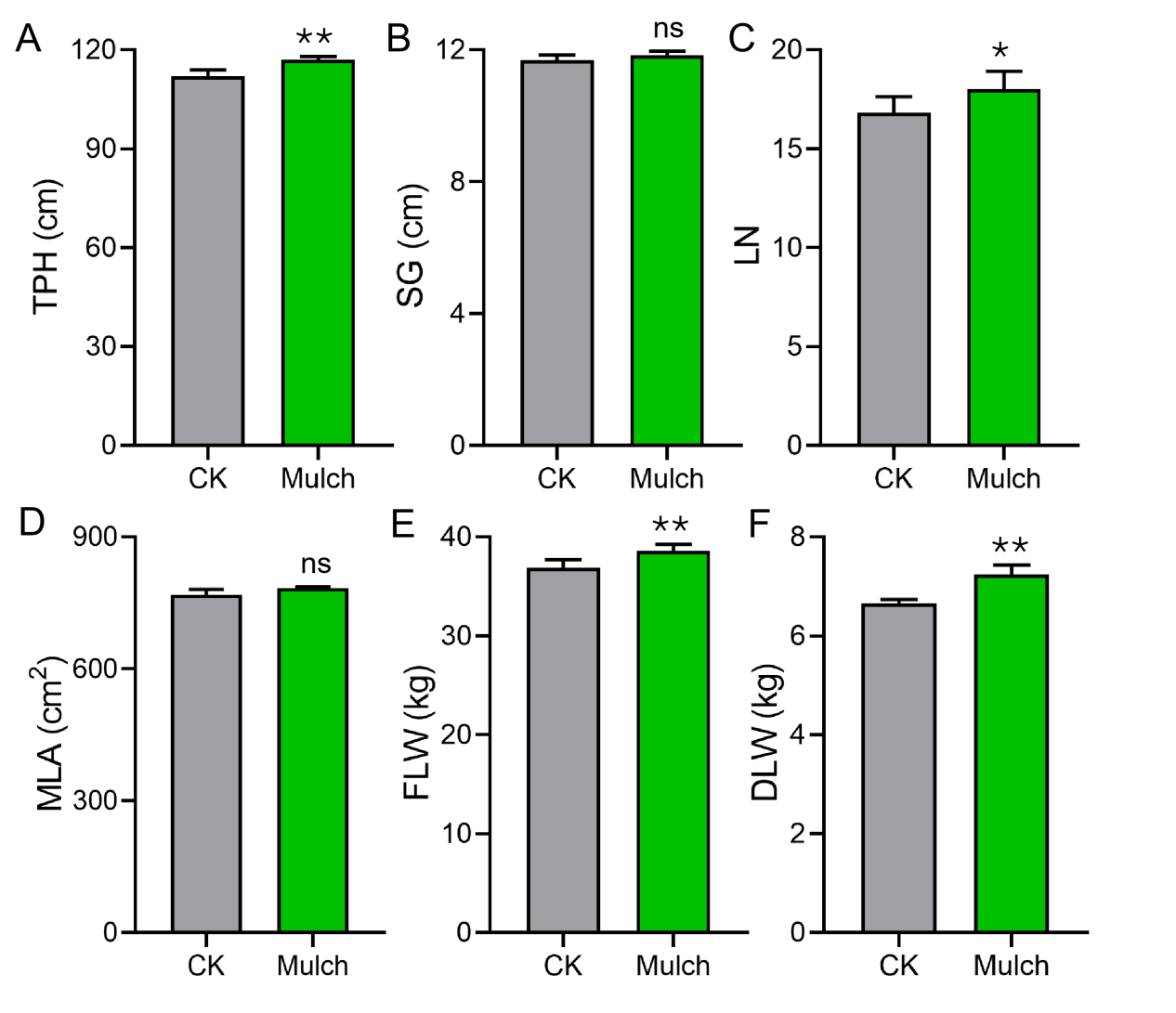
Supplementary Figure 1. Effects of film mulching treatment on agronomic characters of tobacco plants. (A) TPH: tobacco plant height. (B) SG: stem girth. (C) LN: leaf number. (D) MLA: maximum leaf area. (E) FLW: fresh leaf weight. (F) DLW: dry leaf weight. Error bars represent the standard deviations; ns indicates *p* > 0.05, ^🞱^ indicates *p <* 0.05, and ^🞱🞱^ indicates *p <* 0.01 compared to the CK.


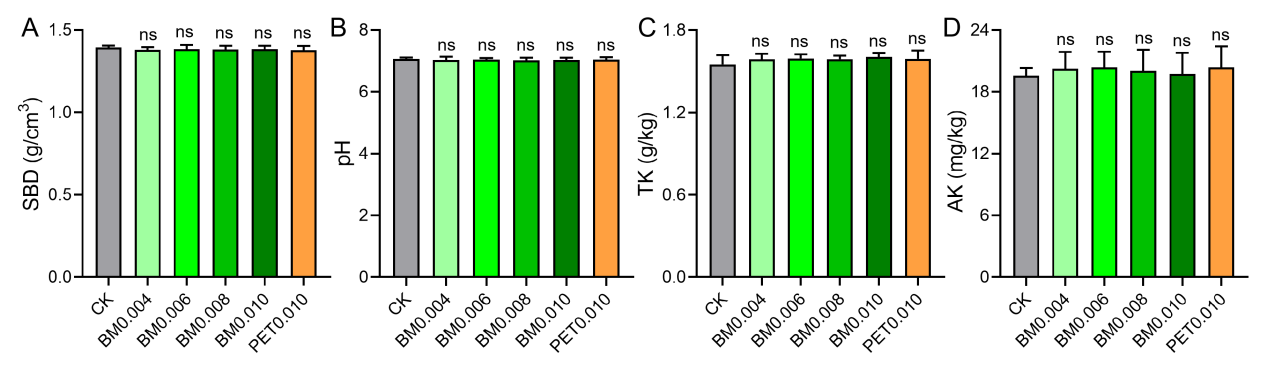


Supplementary Figure 2. Effects of different MF treatments on soil properties. (A) SBD: soil bulk density. (B) pH. (C) TK: total potassium. (D) AK: available potassium. Error bars represent the standard deviations; ns indicates *p >* 0.05 compared to the CK.


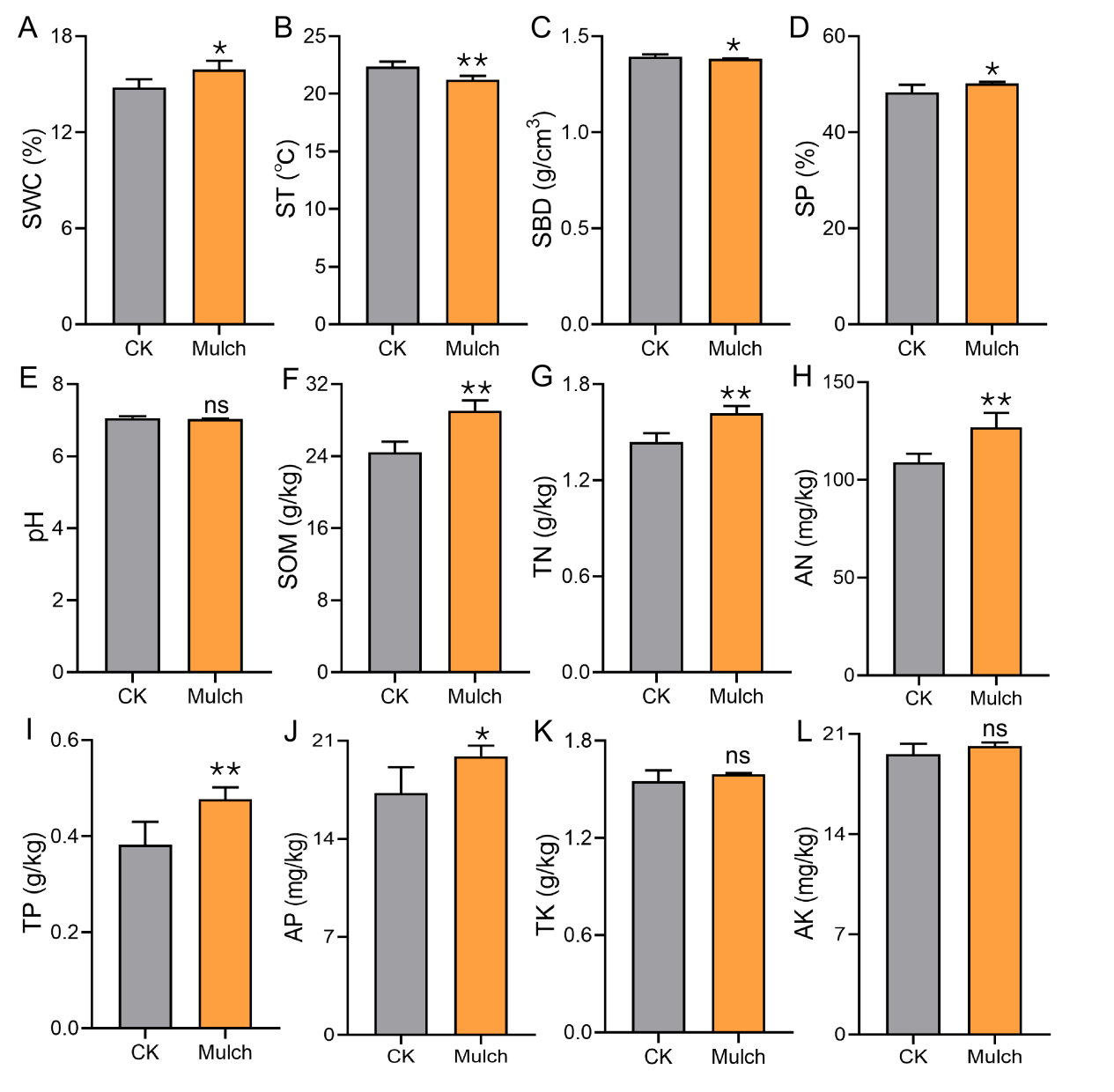


Supplementary Figure 3. Effects of film mulching treatment on soil properties. (A) SWC: soil water content. (B) ST: soil temperature. (C) SBD: soil bulk density. (D) SP: soil porosity. (E) pH. (F) SOM: soil organic matter. (G) TN: total nitrogen. (H) AN: available nitrogen. (I) TP: total phosphorus. (J) AP: available phosphorus. (K) TK: total potassium. (L) AK: available potassium. Error bars represent the standard deviations; ns indicates *p* > 0.05, ^🞱^ indicates *p* < 0.05, and ^🞱🞱^ indicates *p <* 0.01 compared to the CK.

Supplementary Table 1. Correlation analysis between film thickness and agronomic traits, soil properties and soil microorganisms

| Agronomic characters | TPH | SG | LN | MLA | FLW | DLW |
| --- | --- | --- | --- | --- | --- | --- |
|  | 0.657 | 0.473 | 0.662 | 0.571 | 0.640 | 0.842 |
| Soil properties | SWC | ST | SBD | SP | pH | SOM |
|  | 0.716 | -0.782 | -0.192 | 0.616 | -0.097 | 0.723 |
|  | TN | AN | TP | AP | TK | AK |
|  | 0.675 | 0.838 | 0.551 | 0.748 | 0.332 | 0.07 |
| Soil microorganisms | *Sphingomonas* | *Bradyrhizobium* | *Nitrospira* | *Massilia* |  |  |
|  | -0.767 | 0.427 | 0.858 | -0.796 |  |  |

Note: Data are provided as PCC value; the PCC value is calculated via the Pearson correlation method. TPH: tobacco plant height; SG: stem girth; LN: leaf number; MLA: maximum leaf area; FLW: fresh leaf weight; DLW: dry leaf weight; SWC: soil water content; ST: soil temperature; SBD: soil bulk density; SP: soil porosity; SOM: soil organic matter; TN: total nitrogen; AN: available nitrogen; TP: total phosphorus; AP: available phosphorus; TK: total potassium; AK: available potassium.

Supplementary Table 2. The classified bacterial taxa with significant differences in soil abundance among different treatments.

| **Class** | **CK** | **BM004** | **BM006** | **BM008** | **BM010** | **PET010** |
| --- | --- | --- | --- | --- | --- | --- |
| *Alphaproteobacteria* | 0.265±0.033a | 0.255±0.043ab | 0.165±0.030bc | 0.267±0.023a | 0.170±0.048abc | 0.145±0.037c |
| *Gammaproteobacteria* | 0.133±0.005a | 0.174±0.071a | 0.172±0.045a | 0.230±0.057a | 0.212±0.016a | 0.212±0.016a |
| *Bacteroidia* | 0.093±0.016a | 0.072±0.033a | 0.127±0.026a | 0.066±0.015a | 0.108±0.031a | 0.122±0.038a |
| *Acidobacteriia* | 0.197±0.051a | 0.203±0.050a | 0.013±0.005b | 0.137±0.022a | 0.011±0.001b | 0.014±0.006b |
| *Verrucomicrobiae* | 0.027±0.012c | 0.042±0.016bc | 0.107±0.020a | 0.042±0.010bc | 0.089±0.010bc | 0.068±0.040abc |
| *Deltaproteobacteria* | 0.014±0.003d | 0.035±0.007c | 0.066±0.009ab | 0.046±0.005bc | 0.070±0.003a | 0.084±0.012a |
| *Gemmatimonadetes* | 0.030±0.003b | 0.014±0.004b | 0.076±0.013a | 0.031±0.005b | 0.074±0.004a | 0.072±0.012a |
| *Phycisphaerae* | 0.031±0.005a | 0.019±0.004a | 0.035±0.008a | 0.018±0.003a | 0.033±0.018a | 0.031±0.002a |
| *Subgroup_6* | 0.002±0.001b | 0.005±0.002b | 0.049±0.009a | 0.009±0.004b | 0.041±0.004a | 0.040±0.009a |
| *Planctomycetacia* | 0.011±0.005a | 0.011±0.002a | 0.031±0.001a | 0.015±0.008a | 0.023±0.008a | 0.031±0.014a |
| Others | 0.198±0.027a | 0.170±0.014a | 0.160±0.028a | 0.139±0.029a | 0.170±0.012a | 0.187±0.020a |
| **family** | **CK** | **BM004** | **BM006** | **BM008** | **BM010** | **PET010** |
| *Sphingomonadaceae* | 0.159±0.030a | 0.087±0.020ab | 0.109±0.029ab | 0.090±0.024ab | 0.087±0.031ab | 0.067±0.035b |
| *Chitinophagaceae* | 0.076±0.015a | 0.052±0.026a | 0.114±0.025a | 0.054±0.011a | 0.095±0.028a | 0.106±0.031a |
| *Burkholderiaceae* | 0.090±0.011a | 0.079±0.032a | 0.054±0.006a | 0.079±0.021a | 0.061±0.004a | 0.053±0.008a |
| *Gemmatimonadaceae* | 0.030±0.003b | 0.014±0.004b | 0.076±0.013a | 0.031±0.005b | 0.074±0.004a | 0.072±0.012a |
| *Xanthobacteraceae* | 0.050±0.014ab | 0.071±0.013a | 0.016±0.001c | 0.064±0.009a | 0.025±0.010bc | 0.022±0.006c |
| *Acidobacteriaceae_Subgroup_1* | 0.0819±0.0172a | 0.0803±0.0147a | 0.0002±0.0001c | 0.0466±0.0035b | 0.0008±0.0006c | 0.0009±0.0003c |
| *uncultured_bacterium_o_Acidobacteriales* | 0.0671±0.0210a | 0.0718±0.0157a | 0.0007±0.0002b | 0.0546±0.0112a | 0.0016±0.0011b | 0.0015±0.0015b |
| *Chthoniobacteraceae* | 0.011±0.006b | 0.011±0.010b | 0.050±0.014a | 0.008±0.003b | 0.049±0.009a | 0.036±0.025ab |
| *Pedosphaeraceae* | 0.011±0.006c | 0.019±0.004bc | 0.050±0.006a | 0.020±0.004bc | 0.033±0.001ab | 0.026±0.031bc |
| *WD2101_soil_group* | 0.030±0.005a | 0.019±0.004a | 0.031±0.008a | 0.017±0.004a | 0.029±0.016a | 0.026±0.002a |
| Others | 0.395±0.015b | 0.496±0.038ab | 0.496±0.029a | 0.537±0.008a | 0.546±0.014a | 0.591±0.060a |
| **Genus** | **CK** | **BM004** | **BM006** | **BM008** | **BM010** | **PET010** |
| *Sphingomonas* | 0.157±0.029a | 0.078±0.024b | 0.094±0.029ab | 0.073±0.018b | 0.065±0.020b | 0.046±0.039b |
| *Flavisolibacter* | 0.019±0.005bc | 0.011±0.010c | 0.066±0.017a | 0.017±0.005c | 0.040±0.018abc | 0.050±0.010ab |
| *uncultured_bacterium_o_Acidobacteriales* | 0.0671±0.0210a | 0.0718±0.0157a | 0.0007±0.0002b | 0.0546±0.0112a | 0.0016±0.0011b | 0.0015±0.0015b |
| *uncultured_bacterium_f_Gemmatimonadaceae* | 0.007±0.002b | 0.005±0.003b | 0.043±0.009a | 0.009±0.002b | 0.042±0.004a | 0.043±0.005a |
| *uncultured_bacterium_f_WD2101_soil_group* | 0.030±0.004a | 0.019±0.004a | 0.030±0.008a | 0.017±0.003a | 0.028±0.016a | 0.026±0.002a |
| *uncultured_bacterium_c_Subgroup_6* | 0.002±0.001b | 0.005±0.002b | 0.049±0.009a | 0.009±0.004b | 0.041±0.004a | 0.040±0.009a |
| *Candidatus_Udaeobacter* | 0.010±0.005bc | 0.009±0.010c | 0.042±0.013a | 0.006±0.002c | 0.041±0.007ab | 0.031±0.021abc |
| *Gemmatimonas* | 0.022±0.003ab | 0.009±0.001b | 0.029±0.004a | 0.021±0.005ab | 0.029±0.006a | 0.026±0.008a |
| *Bradyrhizobium* | 0.0282±0.0059a | 0.0399±0.0104a | 0.0063±0.0004b | 0.0316±0.0083a | 0.008±0.0043b | 0.0075±0.0042b |
| *uncultured_bacterium_f_SC-I-84* | 0.0021±0.0005b | 0.0029±0.0020b | 0.0330±0.0088a | 0.0052±0.0030b | 0.0376±0.0061a | 0.0379±0.0060a |
| Others | 0.657±0.022ab | 0.750±0.049a | 0.607±0.022b | 0.757±0.010a | 0.667±0.022ab | 0.692±0.075ab |
| **Order** | **CK** | **BM004** | **BM006** | **BM008** | **BM010** | **PET010** |
| *Betaproteobacteriales* | 0.086±0.006b | 0.076±0.030b | 0.133±0.019ab | 0.117±0.029ab | 0.155±0.020a | 0147±0.019a |
| *Sphingomonadales* | 0.159±0.030a | 0.087±0.020ab | 0.109±0.029ab | 0.090±0.024ab | 0.087±0.031ab | 0.067±0.035b |
| *Chitinophagales* | 0.076±0.015a | 0.052±0.026a | 0.117±0.026a | 0.054±0.011a | 0.096±0.030a | 0.108±0.033a |
| *Acidobacteriales* | 0.154±0.037a | 0.156±0.029a | 0.003±0.001b | 0.104±0.010a | 0.005±0.002b | 0.007±0.004b |
| *Rhizobiales* | 0.063±0.014b | 0.101±0.017a | 0.025±0.003c | 0.106±0.006a | 0.045±0.017bc | 0.042±0.014bc |
| *Gemmatimonadales* | 0.030±0.003b | 0.014±0.004b | 0.076±0.013a | 0.031±0.005b | 0.074±0.004a | 0.072±0.012a |
| *Chthoniobacterales* | 0.014±0.008a | 0.017±0.015a | 0.050±0.014a | 0.012±0.004a | 0.049±0.009a | 0.036±0.025a |
| *Myxococcales* | 0.012±0.003c | 0.031±0.007abc | 0.021±0.003bc | 0.044±0.005a | 0.031±0.003abc | 0.032±0.014ab |
| *Xanthomonadales* | 0.010±0.001a | 0.029±0.024a | 0.020±0.013a | 0.046±0.021a | 0.035±0.014a | 0.025±0.005a |
| *Pedosphaerales* | 0.011±0.006c | 0.019±0.004bc | 0.050±0.006a | 0.020±0.004bc | 0.033±0.001ab | 0.026±0.013bc |
| Others | 0.385±0.020a | 0.417±0.016a | 0.395±0.022a | 0.377±0.032a | 0.391±0.013a | 0.438±0.038a |
| **Phylum** | **CK** | **BM004** | **BM006** | **BM008** | **BM010** | **PET010** |
| *Proteobacteria* | 0.412±0.032b | 0.464±0.040ab | 0.402±0.052b | 0.543±0.058a | 0.452±0.047ab | 0.436±0.041ab |
| *Acidobacteria* | 0.203±0.050ab | 0.215±0.052a | 0.120±0.025bc | 0.155±0.026abc | 0.096±0.007c | 0.102±0.013c |
| *Bacteroidetes* | 0.093±0.016a | 0.072±0.033a | 0.128±0.026a | 0.066±0.015a | 0.108±0.031a | 0.124±0.038a |
| *Verrucomicrobia* | 0.027±0.012c | 0.042±0.016bc | 0.107±0.020a | 0.042±0.010bc | 0.089±0.010bc | 0.068±0.040abc |
| *Gemmatimonadetes* | 0.030±0.003b | 0.015±0.006b | 0.084±0.015a | 0.037±0.006b | 0.082±0.006a | 0.082±0.015a |
| *Planctomycetes* | 0.042±0.006a | 0.033±0.006a | 0.068±0.017a | 0.035±0.009a | 0.060±0.027a | 0.069±0.020a |
| *Actinobacteria* | 0.069±0.018a | 0.052±0.012ab | 0.016±0.007c | 0.035±0.015bc | 0.027±0.010bc | 0.017±0.005c |
| *Chloroflexi* | 0.054±0.016a | 0.045±0.016ab | 0.016±0.003b | 0.033±0.014ab | 0.022±0.002b | 0.021±0.003b |
| *Nitrospirae* | 0.005±0.002b | 0.007±0.006b | 0.023±0.005ab | 0.005±0.002b | 0.029±0.006a | 0.039±0.014a |
| *Armatimonadetes* | 0.021±0.004a | 0.015±0.005ab | 0.010±0.003b | 0.008±0.004b | 0.010±0.003b | 0.013±0.002ab |
| Others | 0.045±0.010a | 0.041±0.012a | 0.026±0.009a | 0.041±0.001a | 0.026±0.006a | 0.031±0.009a |

Note: Data are provided as means±SDs; different lowercase letters in the same row indicate signifificant differences (*p* < 0.05).
