# Supplementary figures and images for: Effects of mulch films with different thicknesses on the microbial community of tobacco rhizosphere soil in Yunnan laterite
Source: Front Microbiol. 2024 Sep 23;15:1458470. doi: 10.3389/fmicb.2024.1458470 (PMC11456438; doi:10.3389/fmicb.2024.1458470)

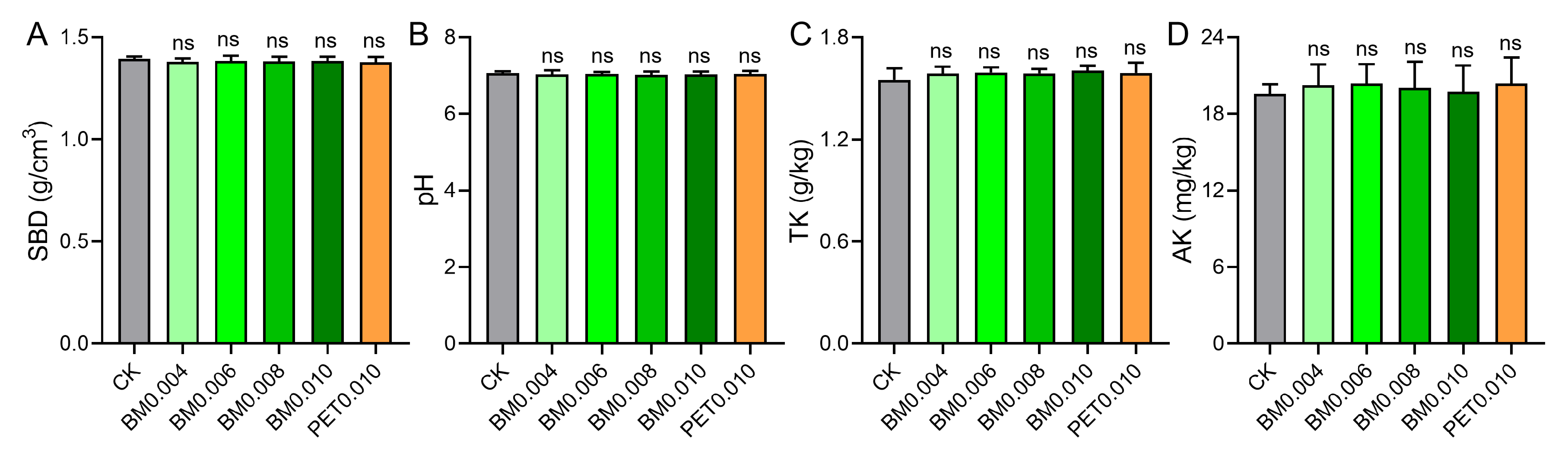

Supplement: Supplementary file 1 [file Data_Sheet_1.zip › Figure-S2.tif]
